# Behavioral Phenotypes in Electronic Health Record Use by Primary Care Providers: a Cluster Analysis

**DOI:** 10.1007/s11606-025-09670-9

**Published:** 2025-07-10

**Authors:** Katharina Tabea Jungo, Niteesh K. Choudhry, John A. Zambrano, Thomas Isaac, Nancy Haff, Julie C. Lauffenburger

**Affiliations:** 1 Center for Healthcare Delivery Sciences (C4HDS), Department of Medicine, Brigham and Women’s Hospital and Harvard Medical School, Boston, MA USA; 2 Division of Pharmacoepidemiology and Pharmacoeconomics, Department of Medicine, Brigham and Women’s Hospital and Harvard Medical School, Boston, MA USA; 3 Institute of Primary Health Care (BIHAM), University of Bern, Bern, Switzerland; 4 Department of Internal Medicine, Atrius Health, Newton, MA USA

**Keywords:** K-means clustering, Cluster analysis, Primary care provider, Electronic health records, EHR use pattern, EHR data

## Abstract

**Background:**

The use of electronic health record (EHR) systems varies among primary care providers (PCPs). However, little is known about how numerous different EHR use behaviors, such as time spent and collaboration in the EHR, cluster together. Prior efforts to quantify characteristics of PCPs using EHRs have generally focused on single behaviors.

**Objective:**

To identify patterns of EHR use among PCPs using a data-driven clustering approach.

**Design:**

Cross-sectional study analyzing EHR data from the 2021 calendar year.

**Participants:**

Primary care providers practicing in a large Massachusetts healthcare system.

**Approach:**

PCPs were assigned to groups based on patterns of EHR use across 30 monthly variables from EHR data using a k-means clustering approach. We used Elbow, Silhouette, and Gap statistic methods to determine the number of clusters. Cluster characteristics were analyzed descriptively.

**Key Results:**

In total, 163 PCPs were included; 103 (63%) PCPs were female, and 113 (69%) were White. Three distinct clusters of PCPs were identified, named based on the EHR characteristics that differed most across the clusters: (1) “High-engagement users”: 38% of PCPs; (2) “Low-engagement users”: 42%; and (3) “Moderate and selective users”: 20%.

**Conclusions:**

This study identified three distinct patterns of EHR use among PCPs, characterized by different levels of engagement with EHR functionality and time spent in the EHR. Further studies are needed to explore how EHR-based interventions could be tailored to different provider workflow styles.

**Supplementary Information:**

The online version contains supplementary material available at 10.1007/s11606-025-09670-9.

## INTRODUCTION

The use of electronic health record (EHR) systems is now widespread.^1^ EHRs have demonstrated benefits for patient care and health systems including reduced medical errors, enhanced quality reporting and facilitated billing.^2^ However, the use of EHR tools has also been linked to disruptions in clinical workflows and increased documentation burden.^2–4^ Physicians spend more than a third of their work time in the EHR,^5–7^ which has been associated with high rates of burnout.^8–16^

EHR use data, derived from “back-end data,” enables the analysis of usage patterns and variability across users, including how long physicians use EHR systems, which parts they interact with, and how administrative tasks affect their time.^17^ Such data have previously been used to gain a better understanding of various aspects of primary care providers’ (PCPs) EHR use.^18–21^ PCPs vary in their use of the EHR,^22–26^ including total time spent in the EHR,^27,28^ frequency of access after regular work hours,^29^ and utilization of tools designed to facilitate documentation.^18^ Lower EHR proficiency, defined as the use of fewer EHR tools that facilitate workflow, documentation, and ordering, and greater note length are associated with more time spent in the EHR.^19,30^ For example, physicians with longer notes spend 39% more time in the EHR after hours and close fewer visits on the same day.^19^ Multiple factors, such as gender and practice setting, have been associated with how the EHR is used by physicians; for example, female physicians spend more time on documentation than male physicians.^27,31–34^ Greater collaboration, such as a higher number of orders placed with team contributions, was associated with less time spent in the EHR.^27,30^ Understanding the variability in EHR use among PCPs can help identify patterns in physician work habits and provide valuable insights for improving clinical practice and freeing up time for patient care.

Prior studies on how PCPs use EHR systems have generally focused on single features, like after-hours use or note composition strategies.^18,35^ Less is known about how PCPs’ EHR use behaviors may cluster together, which may be ultimately more useful for the design of EHR-based interventions. It is critical that EHR-based digital health interventions improve care and reduce provider burden, and a better understanding of PCPs’ EHR use patterns may provide an avenue for designing more personalized and effective interventions. By identifying distinct clusters of EHR usage patterns, tailored strategies for training, support, and workflow optimization can be developed to better address clinicians’ needs. Thus, our goal was to study (a) how primary care providers (PCPs) practicing in a large integrated delivery network cluster into EHR-use phenotypes using a k-means clustering approach, and (b) the characteristics and defining features of these phenotypes. Additionally, we explored which PCP-level characteristics are associated with each phenotype.

## METHODS

### Data source, Population, and Variables

**Figure 1:**
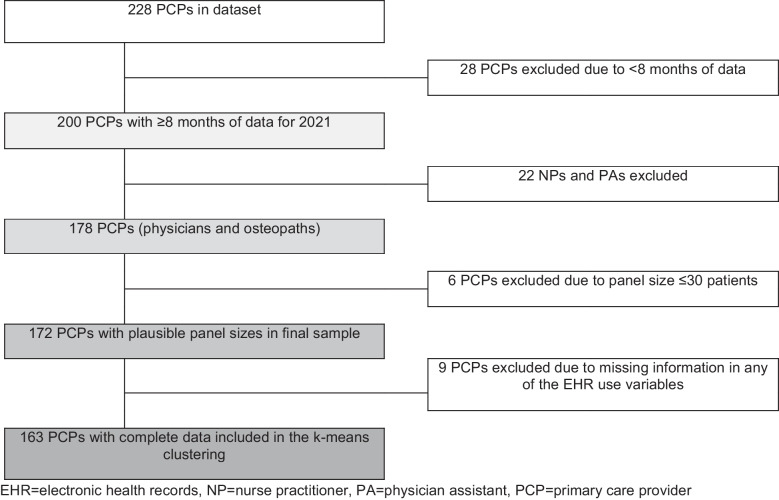
**Consort flow chart.**

We used 2021 calendar year data on PCP sociodemographic characteristics and PCPs’ use of electronic health records from 31 practices across Atrius Health, a large integrated delivery network in Massachusetts. We identified PCPs based on the following eligibility criteria: (1) practicing physicians in the health system in 2021 (excluding nurse practitioners and physician assistants), (2) EHR use data available from ≥ 8 reporting periods (each spanning 28–35 days), (3) a panel size of > 30 patients, and (4) no missing data for EHR use variables (Fig. 1). The present study is a secondary data analysis of the NUDGE trial approved by the Mass General Brigham institutional review board (2019P002167).^36^

The EHR use variables were drawn from EHR use and telemedicine data, which are performance metrics providing information on user workflows, system usage, and operational efficiency.^37^ When selecting variables for the k-means clustering, our goal was to capture key domains of EHR use, including documentation and note-writing, inbox management and messaging, order entry, decision support utilization, time spent in the EHR on days with scheduled appointments and unscheduled days, engagement in various EHR activities, and use of EHR-integrated telemedicine features. We aimed to contribute to the literature by incorporating a broader range of variables measured over a longer period, in contrast to previous studies that focused on a single or small selection of EHR use features (e.g., time spent in the EHR).^18,20,38,39^

To compare across PCPs’ workload, the EHR use variables were normalized for time (e.g., number of days with scheduled appointments or days on which PCPs accessed the EHR system, as provided by the healthcare system). Since the EHR use variables are reported in 28–35-day intervals that approximately correspond to calendar months, we calculated the average of “monthly” values by summing the data from all 12 reporting periods in 2021 and dividing by twelve (eTable 1). We selected the variables included in the clustering approach based on published literature, available structured variables for research purposes, and subject matter expertise.^18,27–29^ Extent of telemedicine use was also included because it reflects how PCPs engaged with the EHR system and their general work practices.

The study variables encompassed two main areas. Part A included PCP and patient characteristics that were not used to describe clusters but not included in the k-means clustering, such as the number of days with appointments per “monthly” reporting period, appointments per day, clinical and administrative full-time equivalent status in 2021, years within the healthcare system, provider type, specialty, gender, race/ethnicity, patient panel size, patient age, and the number of problems on patient problem lists. Part B focused on EHR use data incorporated into k-means clustering, categorized into general work practices (e.g., percentage of days among EHR login days, same-day documentation, orders, notes, messages, and telemedicine visits), collaborative work practices (e.g., shared orders and notes), time spent in the EHR (e.g., minutes spent on various tasks), and the use of specific EHR features (e.g., notes written with EHR shortcuts, predefined phrases) (Table 1).

**Table 1:** List of Variables Included in the k-means Clustering, Including Definitions of EHR Features

Part A. Variables describing PCP and patient characteristics (not included in k-means clustering)
1. Days with scheduled appointments per reporting period 2. Number of appointments per day with scheduled appointments 3. Clinical full-time equivalent in 2021^1^ 4. Admin full-time equivalent in 2021^2^ 5. Years of experience working within the healthcare system 6. Provider type (Osteopath, Physician) 7. Specialty (Family Medicine, Internal Medicine) 8. Gender (Female, Male) 9. Race/Ethnicity (Asian, Black, Hispanic, Multiple, White) 10. Patient panel size in 2021 11. Use of virtual scribes 12. Average patient age 13. Average number of problems on patients’ problem lists
Part B. Variables describing PCPs’ EHR use (included in k-means clustering)
**General work practices** 1. Average percentage of days with scheduled appointments per reporting period 2. Average percentage of patient encounters fully documented and finalized within the same calendar day 3. Average number of orders placed per day with scheduled appointments 4. Average number of notes written per day with scheduled appointments 5. Average number of messages received per day with scheduled appointments 6. Average percentage of telemedicine visits
**Collaborative work practices** 7. Average percentage of orders placed with contributions from other healthcare providers than the PCP 8. Average percentage of notes written with other sources (e.g., other healthcare providers)
**Time spent in the EHR** 9. Average number of minutes in EHR system outside scheduled hours per day with scheduled appointments 10. Average number of minutes in notes per appointment 11. Average number of minutes in notes per day on which PCPs accessed the EHR system 12. Average number of minutes in inbox per appointment^3^ 13. Average number of minutes in inbox per day on which PCPs accessed the EHR system^3^ 14. Average number of minutes in orders per appointment 15. Average number of minutes in orders per day on which PCPs accessed the EHR system 16. Average number of minutes in clinical review per day on which PCPs accessed the EHR system^4^ 17. Average number of minutes in clinical review per appointment 18. Average number of minutes in visit management tool per day on which PCPs accessed the EHR system^5^ 19. Average number of minutes in EHR on unscheduled days (per day) 20. Average number of minutes accessing the EHR system per day outside of 7 am to 7 pm 21. Average turnaround time (in days) for prescription authorization 22. Average turnaround time (in days) for obtaining medical advice upon request
**Variables describing the use of specific EHR features** 23. Average percentage of notes written per reporting period using different tools: • using EHR shortcuts^6^ • using copy-paste • using clinical note editor^7^ • written manually 24. Average percentage of orders placed using order templates & predefined order sets^8^ per reporting period 25. Average number of action shortcuts^9^ used per day on which PCPs accessed the EHR system 26. Average number of predefined phrases^10^ created by provider per reporting period 27. Average number of quick diagnosis buttons^11^ created by provider per reporting period

^1^One full-time equivalent is the workload of one full-time physician

^2^Admin full-time equivalent corresponds to PCPs’ non-clinical workload within the health system

^3^Message center; secure platform for receiving and sending messages

^4^Clinical review: process of examining and assessing clinical information within a patient’s electronic record

^5^Visit management tool: structured, customizable workflow tool that guides healthcare providers through the various tasks and documentation steps required during a patient visit

^6^Features that enhance documentation efficiency by enabling quick insertion of predefined text, data, or templates into notes or records

^7^Documentation tool that guides users through structured, template-based note creation

^8^Predefined groups of orders, documentation templates, and other clinical tools that streamline and standardize the management of specific clinical scenarios or conditions

^9^Customizable, one-click shortcuts that automate repetitive tasks

^10^Pre-defined blocks of text or data that can be inserted into a note or documentation with a simple trigger phrase or shortcut

^11^User-defined, customizable quick access buttons

### Analyses and Clustering Approach

We first used descriptive statistics to analyze EHR use, sociodemographic characteristics, and clinical practices of PCPs overall in our sample (Table 1). Means and standard deviations were calculated for continuous variables. Frequencies and percentages are presented for categorical variables. Then, we used k-means clustering to identify distinct groups of PCPs based on their EHR use (described in detail in the Methods Appendix). After identifying the clusters, we assigned the cluster assignment to the original EHR use data and we used descriptive statistics to report the sociodemographic and EHR use characteristics of PCPs by cluster. We also analyzed differences across the clusters by calculating the standardized mean differences (SMDs).^40^ After describing EHR use patterns in the clusters, we named the clusters based on these patterns. In secondary analyses, we repeated the k-means clustering approach and description of cluster characteristics stratified by the PCPs’ panel size (“ < mean panel size” and “ ≥ mean panel size”), to stratify by clinical workload. According to our collaborators working within the integrated delivery network, panel size is a more appropriate proxy for workload than full-time equivalent.

Finally, we performed a multinomial regression analysis to explore the association between the cluster assignment and PCPs’ sociodemographic variables and clinical practices that were not used in the k-means clustering. The cluster with the largest number of PCPs was selected as the reference cluster. Variables with high collinearity were excluded, with LASSO methods used to reduce the number of variables.^41^ Regression analyses were conducted in Stata (version 18).^42^

## RESULTS

### PCP Characteristics

From the 228 total PCPs, 28 (12.3%) were excluded for having < 8 reporting periods of data, 22 (9.6%) were excluded for being nurse practitioners and physician assistants, 6 (2.6%) were excluded based on mean panel size ≤ 30 patients, and 9 additional PCPs (3.9%) were excluded for having missing data on included EHR use variables (Fig. 1). Thus, the total sample contained data from 163 PCPs.

The baseline characteristics of PCPs, including their sociodemographic information, clinical work practices, and EHR use, are presented in Table 2. Overall, the PCPs’ mean age was 56 years (SD = 7), 63% were female (*n* = 103), and 69% were White (*n* = 113). On average, PCPs had a mean clinical FTE of 0.73 (SD = 0.20), their mean number of appointments per day was 12 (SD = 3), and their mean panel size was 1879 patients (SD = 634).

**Table 2:** PCP Characteristics

		*n* = 163
Variables describing PCP and patient characteristics *(not included in k-means clustering)*		
Days with scheduled appointments per reporting period, mean % (SD)		49.7% (11.1)
Number of appointments per day with scheduled appointments, mean (SD)		11.8 (2.7)
Clinical full-time equivalent in 2021, mean % (SD)^1^		72.7% (19.9)
Admin full-time equivalent in 2021, mean % (SD)^2^		7.9% (16.5)
Years of experience working within the healthcare system, mean (SD)		13.0 (9.9)
Provider type, *n* (%)	Osteopath	6 (3.7)
	Physician	157 (96.4)
Specialty, *n* (%)	Family Medicine	19 (11.7)
	Internal Medicine	144 (88.4)
Gender, *n* (%)	Female	103 (63.2)
	Male	60 (36.8)
Race/ethnicity, *n* (%)	Asian	41 (25.2)
	Black	1 (0.6)
	Hispanic	7 (4.3)
	Multiple	1 (0.6)
	White	113 (69.3)
Panel size in 2021, mean (SD)		1879 (634)
Any use of virtual scribes, *n* (%)		19 (11.4%)
Patient age (years), mean (SD)		55.7 (6.7)
Number of problems on patients’ problem lists, mean (SD)		11.8 (3.2)
**Variables describing PCPs’ EHR use *(included in k-means clustering)***		
**General work practices**		
Percentage of days with appointments among days logged into the EHR, mean (SD)		65.0 (15)
Percentage of patient encounters fully documented and finalized within the same calendar day, mean (SD)		76.2% (26.3)
Number of orders placed per day with scheduled appointments, mean (SD)		103.9 (31.4)
Number of notes written per day with scheduled appointments, mean (SD)		84.3 (31.1)
Number of messages received per day with scheduled appointments, mean (SD)		80.2 (25.0)
Percentage of telemedicine visits, mean (SD)		21.1% (11.3)
**Collaborative work practices**		
Percentage of orders placed with contributions from other healthcare providers than the PCP, mean (SD)		8.4% (10.1)
Percentage of notes written with other sources (e.g., other healthcare providers), mean (SD)		7.1% (18.6)
**Time spent in the EHR**		
Number of minutes in EHR system outside scheduled hours per day with scheduled appointments, mean (SD)		67.2 (43.5)
Number of minutes in notes per appointment, mean (SD)		8.8 (4.5)
Number of minutes in notes per day on which PCPs accessed the EHR system, mean (SD)		63.3 (30.5)
Number of minutes in inbox per appointment,^3^ mean (SD)		5.6 (2.7)
Number of minutes in inbox per day on which PCPs accessed the EHR system,^3^ mean (SD)		38.6 (14.0)
Number of minutes in orders per appointment, mean (SD)		4.0 (1.3)
Number of minutes in orders per day on which PCPs accessed the EHR system, mean (SD)		29.4 (10.2)
Number of minutes in clinical review per day on which PCPs accessed the EHR system,^4^ mean (SD)		37.1 (17.7)
Number of minutes in clinical review per appointment, mean (SD)		5.2 (2.6)
Number of minutes in visit management tool per day on which PCPs accessed the EHR system,^5^ mean (SD)		12.0 (5.2)
Number of minutes in EHR on unscheduled days (per day)**,** mean (SD)		78.9 (49.5)
Number of minutes accessing the EHR system per day outside of 7 am to 7 pm, mean (SD)		37.4 (24.7)
Turnaround time in days—prescription authorization, mean (SD)		0.7 (2.1)
Turnaround time in days—request for obtaining medical advice, mean (SD)		4.1 (11.7)
**Use of specific EHR features**		
Percentage of notes written per reporting period using different tools, mean (SD)	EHR shortcuts^6^	65.5 (14.2)
	Copy-paste	5.5 (8.9)
	Clinical note editor^7^	1.5 (4.5)
	Manually	17.7 (12.8)
Percentage of order from order templates & predefined order sets^8^ per reporting period, mean (SD)		86.1 (3.7)
Number of action shortcuts^9^ used per day logged into the EHR, mean (SD)		1.8 (2.6)
Number of predefined phrases^10^ created by provider per reporting period, mean (SD)		184.4 (246.6)
Number of quick diagnosis buttons^11^ created by provider per reporting period, mean (SD)		0.8 (0.4)

There was no missing data

^1^One full-time equivalent is the workload of one full-time physician

^2^Admin full-time equivalent corresponds to PCPs’ non-clinical workload within the health system

^3^Message center; secure platform for receiving and sending messages

^4^Clinical review: process of examining and assessing clinical information within a patient’s electronic record

^5^Visit management tool: structured, customizable workflow tool that guides providers through various tasks required during a patient visit

^6^Features that enable quick insertion of predefined text, data, or templates into notes or records

^7^Documentation tool that guides users through structured, template-based note creation

^8^Predefined groups of orders, documentation templates and other clinical tools that streamline and standardize clinical management

^9^Customizable, one-click shortcuts that automate repetitive tasks

^10^Pre-defined blocks of text or data that can be inserted into a note or documentation with a simple trigger phrase or shortcut

^11^User-defined, customizable quick access buttons

On average, PCPs in our sample had appointments on 65% (SD = 15) of the days they logged into the EHR. Of the patient encounters, 76.2% (SD = 26.3) were fully documented and finalized on the same day. PCPs spent an average of 67.2 min (SD = 43.5 min) per day outside of scheduled hours on days with appointments and 78.9 min (SD = 49.5 min) per day on unscheduled days. Collaborative work practices, including contributions from other healthcare providers, make up a small portion of the workflow, with 8.4% of orders (SD = 10.1) and 7.1% (SD = 18.6) of notes involving other sources (Table 2).

### Clustering

Based on the Elbow method, Silhouette method, and Gap statistics, and confirmed by the 30 indices of the *NbClust* package, the number of optimal clusters was determined to be three for the main analysis with all PCPs (eFigure 1). For the secondary analyses stratified by panel size, k-means clustering was performed with 2 clusters each (“ < mean panel size” and “ ≥ mean panel size”). eTable 1 shows PCPs’ sociodemographic and employment characteristics across clusters with SMDs. Cluster plots confirmed similarities among PCPs assigned to the same cluster (eFigure 2). After describing EHR use patterns in the clusters, we named the clusters based on these patterns.

**Figure 2:**
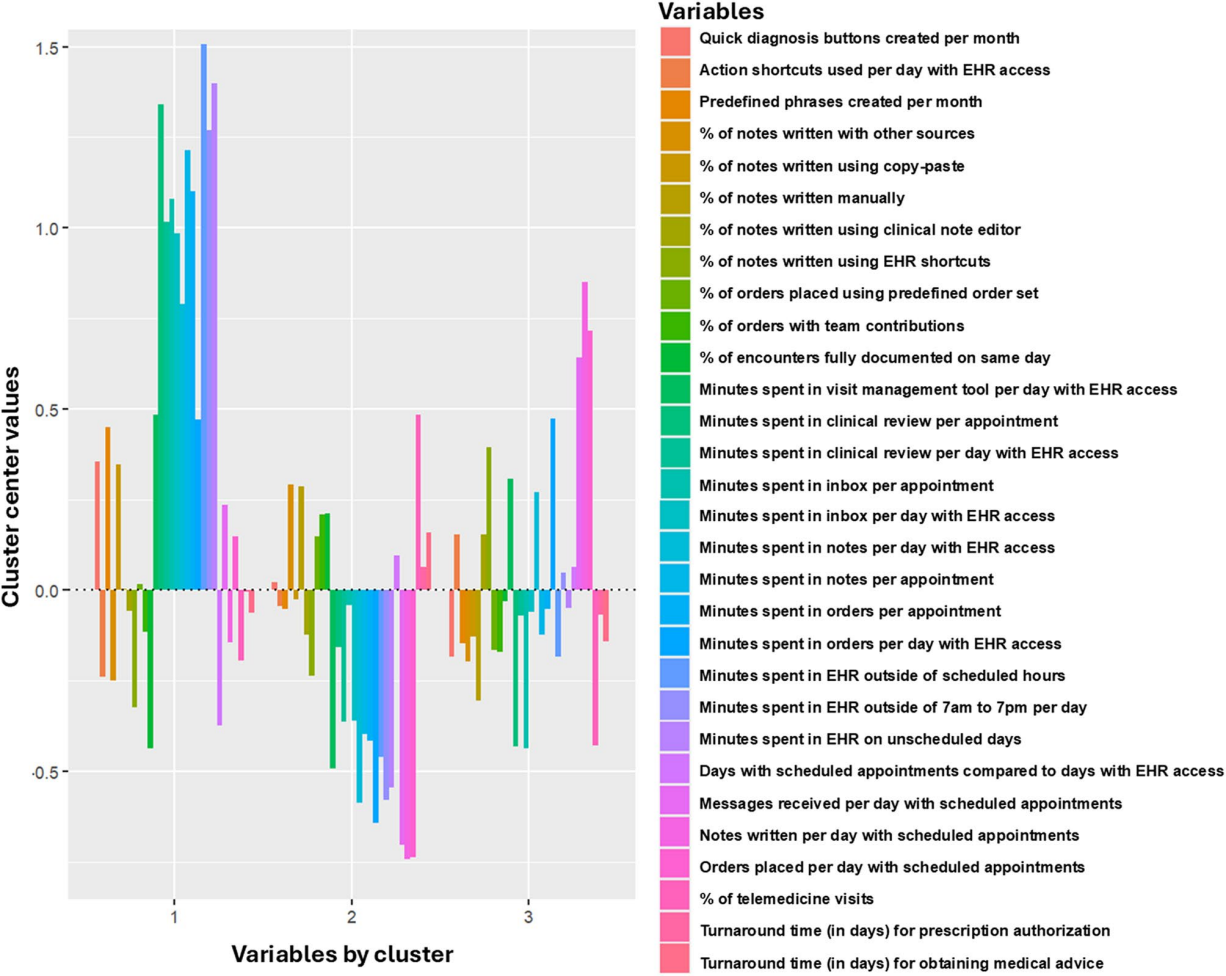
**Cluster centers and distribution of data points across primary care providers’ EHR usage patterns.**

Cluster 1 is characterized by extensive time spent in the EHR across various functions, including frequent use of smart phrases and the creation of quick diagnosis buttons (“High-engagement users”). PCPs in cluster 2 have minimal EHR interaction, with lower turnaround times and less time spent on inbox management, note-writing, and order placement. They also use documentation tools and clinical review features less frequently (“Low-engagement users”). PCPs in cluster 3 exhibit moderate engagement with documentation, order entry, and inbox management. Their use of EHR features is more balanced, without extreme highs or lows across most metrics (“Moderate and selective users”) (Fig. 2). eFigure 3 shows the relative differences in PCPs’ EHR use by cluster for variables with SMD ≥ 0.5 (considered a moderate difference between groups).^43–45^

Of these three clusters, cluster 3 had the highest percentage of female PCPs (72%), and cluster 2 had the highest percentage of non-White PCPs (39%). The percentages of PCPs with a specialization in Internal Medicine were comparable across clusters. PCPs in cluster 1 had the largest clinical FTE and largest mean panel sizes. PCPs in cluster 1 (*n* = 62) had the lowest percentage of telemedicine visits, and the highest number of orders placed, notes written, and messages (named as “High-engagement users” based on these characteristics). PCPs in cluster 2 (*n* = 69) spent least time in the EHR, had the highest percentage of telemedicine visits, and the lowest number of orders placed, notes written, and messages (named as “Low-engagement users”). PCPs in cluster 3 (*n* = 32) spent the greatest amount of time in the EHR (named as “Moderate and selective users”).

### Exploration of Predictors of Cluster Membership

In the multinomial regression analysis, we compared the likelihood of a PCP being in cluster 1 (“High patient load”) or cluster 3 (“Autonomy-driven workflows”), relative to cluster 2 (“Team-centric workflows”) based on PCPs’ sociodemographic and employment characteristics. We selected cluster 2 as the reference group, as it contained the largest number of PCPs. PCPs with administrative duties (RR = 0.33, 95% CI 0.13–0.82 those of non-White race/ethnicity (RR = 0.31, 95% CI 0.14–0.72), and those with more years in the healthcare system (RR per 10-year increase, 0.55; 95% CI 0.36–0.83) were less likely to be in cluster 1 (eTable 3). No PCP characteristics were significantly associated with being assigned to cluster 3 compared to cluster 2.

### Secondary Analyses By Panel Size

eTable 2 shows PCPs’ sociodemographic and employment characteristics across clusters with SMDs. eFigure 4 shows the relative differences in PCPs’ EHR use by cluster from the secondary analysis stratified by panel size. Each stratification had the same two types of clusters, which mirrored clusters 2 and 3 from the main analysis. For instance, PCPs in cluster 1 had a higher number of visits closed on the same day, a higher percentage of telemedicine visits, and a lower number of orders placed, notes written, and messages (“Low-engagement users”). Similar to the main analysis, PCPs in cluster 2 had a higher volume, with more orders placed, notes written, and messages, created more SmartPhases and spent more time in the EHR (“Moderate and selective users”).

## DISCUSSION

In this cross-sectional study of PCPs across a large integrated delivery network, we uncovered three unique clusters of PCPs based on their EHR use behaviors. These differences were largely characterized by different levels of engagement with EHR functionality and how much time they spent in the EHR. These findings highlight the complexity of how PCPs use EHR systems and the potential for tailoring EHR-based interventions based on these differences in behavior.

As hypothesized, we found that PCPs cluster together with regard to their EHR use characteristics. Our findings are in line with the results from a recent analysis that examined six EHR use characteristics across a 1-month period in July 2020 in contrast to ours, which included three times as many variables over a 1-year period.^46^ In this analysis, the authors identified a similar “higher-efficiency, lower-time outside of scheduled hours” cluster and a “lower-efficiency, higher-time outside of scheduled hours” cluster, even when stratified by gender and specialty.^39^ To our knowledge, other studies have focused only on individual EHR use characteristics. For example, one clustering study using an unsupervised clustering approach called affinity propagation looked uniquely at time spent in the EHR and found differences between the amount of time spent in the EHR in different clusters.^38^ They identified two phenotype clusters of physicians with higher-than-average work outside of scheduled hours showed varying EHR usage, with one group working from home out of necessity and the other preferring ad hoc hours, while the remaining two groups included physicians with lower-than-average EHR time and those who spent the most time documenting notes. Like our results, time spent in the EHR and differences in documentation played an important role. Another recently published clustering study using six EHR use variables related to physicians’ workload and inbox management identified two clusters: a high-burden cluster, predominantly consisting of experienced primary care physicians, and a lower-burden cluster, primarily composed of younger specialists.^20^ This study identified significant differences by physician specialty and gender.

The identification of EHR usage clusters and their relationship with PCP demographic characteristics provides opportunities to improve both clinical and administrative aspects of healthcare. By understanding how PCPs interact with EHR systems, administrators and researchers can develop targeted training, support, and interventions tailored to specific needs. These insights can help optimize EHR features, reduce administrative burden, and address gaps in care. For instance, some PCPs may not fully utilize EHR features that flag high-risk patients, missing opportunities for preventive care. Additionally, clustering can reveal patterns of administrative burden, helping to reduce unnecessary tasks and improve workload distribution. If validated in other settings, these clusters could inform the development of personalized user “phenotypes” to guide EHR software design and institutional policies, enhancing both clinician and patient outcomes.

Our results also have direct implications for EHR-based interventions. By targeting specific PCP clusters based on their EHR use patterns, interventions can be aligned with individual workflows, improving feasibility and effectiveness. For example, PCPs with higher volumes of orders may require different intervention delivery strategies than those with lower volumes; for instance, PCPs who order certain tests more frequently would encounter related EHR-based decision support more often. Second, a criticism of prior EHR-based interventions is suboptimal timing (e.g., alerts shown to PCPs after patients have left) or that they have interrupted clinical workflows.^47^ Understanding when PCPs spend time in the EHR (e.g., during or outside scheduled hours, during, before or after visits) presents opportunities for better-aligned alerts and notifications. For example, sending alerts to clinicians who are charting the night before the visit may have unintended effects. Third, our results on PCPs’ EHR use patterns could inform the content and features of EHR-based interventions. For example, PCPs who do more manual data entry might benefit from streamlined workflows or advanced EHR features that reduce manual entry and improve efficiency. Regardless, better tailoring of EHR interventions may be a strategy to improve intervention efficiency and reduce potential burnout and fatigue, improve provider satisfaction with EHR-based interventions, and should be investigated further.

The PCP clusters themselves warrant further consideration. First, cluster 1 “High-engagement users” comprises PCPs who are highly productive in executing many tasks within the EHR, even after accounting for PCP workload (by normalizing the variables, where needed). Conversely, cluster 2 “Low-engagement users” highlights a group of PCPs who spend less time in the EHR and have more team-centric workflows, likely because they use more virtual scribes or have high functioning medical assistants. Prior work has found that having more telemedicine visits is associated with more time spent in the EHR mostly due to documentation,^48^ while time spent on messaging, in notes, and outside of scheduled hours may decrease.^49^ Finally, cluster 3 “Moderate and selective users,” which had less teamwork and spent the most time in the EHR, may reflect a group of PCPs who, while executing fewer “tasks,” engage deeply with patient records, possibly because of meticulous documentation practices, although this is not possible to know from these data alone. However, this hypothesis matches with recent literature showing that PCPs’ note composition strategies influence their time in notes and in the EHR ^18,30^ and that more collaborative work is associated with less time in the EHR.^27,30^ The same study also found that using more shortcuts and predefined phrases was associated with more time spent in the EHR, which seems counterintuitive and is similar to differences we observed between our “High-engagement users” and “Moderate and selective users” clusters, such as for example the number of predefined phrases created.

Time spent in the EHR outside of scheduled hours and on days without appointments has increased since the Covid-19 pandemic.^16^ That sociodemographic characteristics like gender and race/ethnicity were similar across clusters suggests that these factors alone may not drive differences in EHR use, despite some of these factors being individually associated with certain EHR use behaviors (e.g., female PCPs spending more time on documentation and more time in the EHR outside of scheduled hours).^27,31–34,50^ The association between non-White race/ethnicity and being in the “High-engagement users” cluster (cluster 1) suggests that there may be underlying factors like variation in the patient populations that may contribute to these differences, which we were unable to account for in our analyses.

### Limitations

Our study has several limitations. First, we were limited to EHR use variables collected within the integrated healthcare delivery network. We had limited information about PCPs’ sociodemographic characteristics (e.g., provider age) and patient populations, as well as no data on whether PCPs worked with residents or medical students. Additionally, we lacked information on the EHR use culture within the healthcare system and individual practices, which can influence how PCPs use EHR systems. The selection of variables may have influenced our findings; had we picked a different selection of variables, we might not have identified the same clusters. Second, we used data from PCPs within one integrated delivery network in the Boston metropolitan area, which could reduce generalizability.^51^ Third, we used data from 2021, when the COVID-19 pandemic was still happening but had begun to stabilize. This could affect some variables such as the use of telemedicine, though data suggests telemedicine use remains prevalent.^52^ Fourth, while we could have adjusted for PCPs’ workload in different ways, we decided to use the variables adjusted by time variables (e.g., per day on which PCPs accessed the EHR system, as provided by the healthcare system). Finally, our ability to detect associations in the regression analysis may have been limited by the relatively small sample size.

## CONCLUSION

The results of our study highlight the heterogenous nature of EHR use among PCPs and the potential value of using clustering approaches to identify usage patterns across multiple factors. Further studies are needed to explore how EHR-based interventions could be tailored to different provider workflow styles. Future studies should also examine how the clustered nature of PCPs’ EHR use is associated with PCP outcomes (e.g., burnout), quality measures and cost of care, and the implementation of EHR-based interventions.

## Supplementary Information

Below is the link to the electronic supplementary material.

ESM 1 (DOCX 827 KB)

## Data Availability

The data that support the findings of this study are not publicly available due to participant privacy and institutional data sharing restrictions.
