# Supplementary material for: Behavioral Phenotypes in Electronic Health Record Use by Primary Care Providers: a Cluster Analysis
Source: J Gen Intern Med. 2025 Jul 10;40(15):3723–32. doi: 10.1007/s11606-025-09670-9 (PMC12612465; doi:10.1007/s11606-025-09670-9)
Supplement: Supplementary file 1 — (DOCX 827 KB) [file 11606_2025_9670_MOESM1_ESM.docx]

**Supporting Material**

Behavioral Phenotypes in Electronic Health Record Use by Primary Care Providers: a Cluster Analysis

**Authors**

Katharina Tabea Jungo^1,2,3^, Niteesh K Choudhry^1,2^, John A Zambrano^4^, Thomas Isaac^4^, Nancy Haff^1,2^, Julie C Lauffenburger^1,2^

**Affiliations**

^1^ Center for Healthcare Delivery Sciences (C4HDS), Department of Medicine, Brigham and Women's Hospital and Harvard Medical School, Boston, Massachusetts, USA.

^2^ Division of Pharmacoepidemiology and Pharmacoeconomics, Department of Medicine, Brigham and Women's Hospital and Harvard Medical School, Boston, Massachusetts, USA.

^3^ Institute of Primary Health Care (BIHAM), University of Bern, Bern, Switzerland.

^4^ Department of Internal Medicine, Atrius Health, Newton, MA, USA.

**Table of content**

[Methods Appendix 1. kmeans clustering methods. 2](#_Toc196743621)

[eTable 1. Description of variable definitions. 3](#_Toc196743622)

[eFigure 1. Different methods to identify the right number of clusters. 5](#_Toc196743623)

[eFigure 2. Cluster plots. 7](#_Toc196743624)

[eTable 2. PCP characteristics and EHR use by cluster. 8](#_Toc196743625)

[eTable 3. PCP characteristics and EHR use by cluster: Stratified by panel size. 10](#_Toc196743626)

[eFigure 3. Relative differences in PCPs’ EHR use by cluster (*compared to Cluster 2*) 12](#_Toc196743627)

[eFigure 4. Differences in PCPs’ EHR use by cluster: Stratified by panel size (relative differences) 13](#_Toc196743628)

[eTable 4. Multinomial regression results of the associations between PCP characteristics and the identified EHR use clusters (n=163) 14](#_Toc196743629)

## Methods Appendix 1. kmeans clustering methods.

First, we standardized the data by scaling each variable to have a mean of 0 and a standard deviation of 1. Second, we used the Elbow, Silhouette, and Gap statistic methods, three commonly used approaches that combine both statistical considerations and visual interpretations,^1^ to determine the optimal number of clusters (k). The Elbow method visualizes the percentage of variance explained by the clusters as a function of the number of clusters, helping to identify the optimal cluster count by highlighting the point at which the within-cluster sum of squares shows diminishing returns.^1^ This "elbow" point indicates where adding more clusters no longer significantly improves the model. The Silhouette method, on the other hand, assesses the quality of the clustering by measuring both the cohesion (how close data points are within the same cluster) and the separation (how distinct a cluster is from others), providing a quantitative measure of cluster validity.^1^ Lastly, the Gap statistic compares the within-cluster dispersion of the observed data to that of a reference null distribution, ensuring that the identified clusters are not due to random chance, but reflect true patterns in the data.^1^ Together, these methods provide a robust framework for selecting the optimal number of clusters and evaluating the quality and significance of clustering results. In addition to these three commonly used methods, we used the R package *NbClust*, which provides 30 indices for determining the number of clusters, to determine the number of clusters.^2^ Next, we applied the k-means algorithm to the number of clusters identified, specifically using the R package *kmeans*^3^ with 100 iterations and 100 random starts, which optimizes the Euclidean distance by default (**Appendix 1, Part B**). The k-means algorithm iteratively assigns each data point to the nearest cluster center and updates the cluster centers until the algorithm converges.^4^ In other words, k-means clustering partitions a dataset into distinct groups (clusters) by assigning each data point to the nearest cluster center (centroid), iteratively updating the centroids to minimize the within-cluster variance. After convergence, we obtained the final cluster centers, assigned each data point to one of the clusters, and plotted data points grouped into the clusters based on the two first dimensions of a principal component analysis (**eFigure 1**).

## eTable 1. Description of variable definitions.

| **Variable name** | **Definition** |
| --- | --- |
| **General work practices** |  |
| Average percentage of days with scheduled appointments per reporting period | The percentage of days with scheduled appointments per reporting period was reported by the healthcare system for each monthly period, with each period ranging from 28 to 35 days. We calculated the average across all 12 reporting periods in 2012 and divided the total by 12. |
| Average percentage of patient encounters fully documented and finalized within the same calendar day | The percentage of patient encounters fully documented and finalized within the same calendar day was reported by the healthcare system for each monthly period, with each period ranging from 28 to 35 days. We calculated the average across all 12 reporting periods in 2012 and divided the total by 12. |
| Average number of orders placed per day with scheduled appointments | The number of orders placed per day with scheduled appointments was reported by the healthcare system for each monthly period, with each period ranging from 28 to 35 days. We calculated the average across all 12 reporting periods in 2012 and divided the total by 12. |
| Average number of notes written per day with scheduled appointments | The number of notes written per day with scheduled appointments was reported by the healthcare system for each monthly period, with each period ranging from 28 to 35 days. We calculated the average across all 12 reporting periods in 2012 and divided the total by 12. |
| Average number of messages received per day with scheduled appointments | The number of messages received per day with scheduled appointments was reported by the healthcare system for each monthly period, with each period ranging from 28 to 35 days. We calculated the average across all 12 reporting periods in 2012 and divided the total by 12. |
| Average percentage of telemedicine visits | The percentage of telemedicine visits was reported by the healthcare system for each monthly period, with each period ranging from 28 to 35 days. We calculated the average across all 12 reporting periods in 2012 and divided the total by 12. |
| **Collaborative work practices** |  |
| Average percentage of orders placed with contributions from other healthcare providers than the PCP | The percentage of orders placed with contributions from other healthcare providers than the PCP was reported by the healthcare system for each monthly period, with each period ranging from 28 to 35 days. We calculated the average across all 12 reporting periods in 2012 and divided the total by 12. |
| Average percentage of notes written with other sources (e.g., other healthcare providers) | The percentage of notes written with other sources (e.g., other healthcare providers) was reported by the healthcare system for each monthly period, with each period ranging from 28 to 35 days. We calculated the average across all 12 reporting periods in 2012 and divided the total by 12. |
| **Time spent in the EHR** |  |
| Average number of minutes in EHR system outside scheduled hours per day with scheduled appointments | The number of minutes in EHR system outside scheduled hours per day with scheduled appointments was reported by the healthcare system for each monthly period, with each period ranging from 28 to 35 days. We calculated the average across all 12 reporting periods in 2012 and divided the total by 12. |
| Average number of minutes in notes per appointment | The number of minutes in notes per appointment was reported by the healthcare system for each monthly period, with each period ranging from 28 to 35 days. We calculated the average across all 12 reporting periods in 2012 and divided the total by 12. |
| Average number of minutes in notes per day on which PCPs accessed the EHR system | The number of minutes in notes per day on which PCPs accessed the EHR system was reported by the healthcare system for each monthly period, with each period ranging from 28 to 35 days. We calculated the average across all 12 reporting periods in 2012 and divided the total by 12. |
| Average number of minutes in inbox per appointment^3^ | The number of minutes in inbox per appointment was reported by the healthcare system for each monthly period, with each period ranging from 28 to 35 days. We calculated the average across all 12 reporting periods in 2012 and divided the total by 12. |
| Average number of minutes in inbox per day on which PCPs accessed the EHR system^3^ | The number of minutes in inbox per day on which PCPs accessed the EHR system was reported by the healthcare system for each monthly period, with each period ranging from 28 to 35 days. We calculated the average across all 12 reporting periods in 2012 and divided the total by 12. |
| Average number of minutes in orders per appointment | The number of minutes in orders per appointment was reported by the healthcare system for each monthly period, with each period ranging from 28 to 35 days. We calculated the average across all 12 reporting periods in 2012 and divided the total by 12. |
| Average number of minutes in orders per day on which PCPs accessed the EHR system | The number of minutes in orders per day on which PCPs accessed the EHR system was reported by the healthcare system for each monthly period, with each period ranging from 28 to 35 days. We calculated the average across all 12 reporting periods in 2012 and divided the total by 12. |
| Average number of minutes in clinical review per day on which PCPs accessed the EHR system^4^ | The number of minutes in clinical review per day on which PCPs accessed the EHR system was reported by the healthcare system for each monthly period, with each period ranging from 28 to 35 days. We calculated the average across all 12 reporting periods in 2012 and divided the total by 12. |
| Average number of minutes in clinical review per appointment | The number of minutes in clinical review per appointment was reported by the healthcare system for each monthly period, with each period ranging from 28 to 35 days. We calculated the average across all 12 reporting periods in 2012 and divided the total by 12. |
| Average number of minutes in visit management tool per day on which PCPs accessed the EHR system^5^ | The number of minutes in visit management tool per day on which PCPs accessed the EHR system was reported by the healthcare system for each monthly period, with each period ranging from 28 to 35 days. We calculated the average across all 12 reporting periods in 2012 and divided the total by 12. |
| Average number of minutes in EHR on unscheduled days (per day) | The number of minutes in EHR on unscheduled days (per day) was reported by the healthcare system for each monthly period, with each period ranging from 28 to 35 days. We calculated the average across all 12 reporting periods in 2012 and divided the total by 12. |
| Average number of minutes accessing the EHR system per day outside of 7am to 7pm | The number of minutes accessing the EHR system per day outside of 7am to 7pm was reported by the healthcare system for each monthly period, with each period ranging from 28 to 35 days. We calculated the average across all 12 reporting periods in 2012 and divided the total by 12. |
| Average turnaround time (in days) for prescription authorization | The turnaround time (in days) for prescription authorization was reported by the healthcare system for each monthly period, with each period ranging from 28 to 35 days. We calculated the average across all 12 reporting periods in 2012 and divided the total by 12. |
| Average turnaround time (in days) for obtaining medical advice upon request | The turnaround time (in days) for obtaining medical advice upon request was reported by the healthcare system for each monthly period, with each period ranging from 28 to 35 days. We calculated the average across all 12 reporting periods in 2012 and divided the total by 12. |
| **Variables describing the use of specific EHR features** | |
| Average percentage of notes written per reporting period using different tools:   - using EHR shortcuts^6^ - using copy-paste - using clinical note editor^7^ - written manually | The percentage of notes written per reporting period using different tools were reported by the healthcare system for each monthly period, with each period ranging from 28 to 35 days. We calculated the average across all 12 reporting periods in 2012 and divided the total by 12 for each of the tools. |
| Average percentage of orders placed using order templates & predefined order sets^8^ per reporting period | The percentage of orders placed using order templates & predefined order sets^8^ per reporting period was reported by the healthcare system for each monthly period, with each period ranging from 28 to 35 days. We calculated the average across all 12 reporting periods in 2012 and divided the total by 12. |
| Average number of action shortcuts^9^ used per day on which PCPs accessed the EHR system | The number of action shortcuts^9^ used per day on which PCPs accessed the EHR system was reported by the healthcare system for each monthly period, with each period ranging from 28 to 35 days. We calculated the average across all 12 reporting periods in 2012 and divided the total by 12. |
| Average number of predefined phrases^10^ created by provider per reporting period | The number of predefined phrases^10^ created by provider per reporting period was reported by the healthcare system for each monthly period, with each period ranging from 28 to 35 days. We calculated the average across all 12 reporting periods in 2012 and divided the total by 12. |
| Average number of quick diagnosis buttons^11^ created by provider per reporting period | The number of quick diagnosis buttons^11^ created by provider per reporting period was reported by the healthcare system for each monthly period, with each period ranging from 28 to 35 days. We calculated the average across all 12 reporting periods in 2012 and divided the total by 12. |

^1^ One full-time equivalent is the workload of one full-time physician.

^2^ Admin full-time equivalent corresponds to PCPs’ non-clinical workload within the health system.

^3^ Message center; secure platform for receiving and sending messages.

^4^ Clinical review: Process of examining and assessing clinical information within a patient's electronic record.

^5^ Visit management tool: Structured, customizable workflow tool that guides healthcare providers through the various tasks and documentation steps required during a patient visit.

^6^ Features that enhance documentation efficiency by enabling quick insertion of predefined text, data, or templates into notes or records.

^7^ Documentation tool that guides users through structured, template-based note creation.

^8^ Predefined groups of orders, documentation templates, and other clinical tools that streamline and standardize the management of specific clinical scenarios or conditions.

^9^ Customizable, one-click shortcuts that automate repetitive tasks.

^10^ Pre-defined blocks of text or data that can be inserted into a note or documentation with a simple trigger phrase or shortcut.

^11^ User-defined, customizable quick access buttons.

# eFigure 1. Different methods to identify the right number of clusters.

| **All PCPs** | **By panel size** | | |
| --- | --- | --- | --- |
|  | **<mean panel size*** | **≥mean panel size*** | |
| **Elbow method** | | | |
| 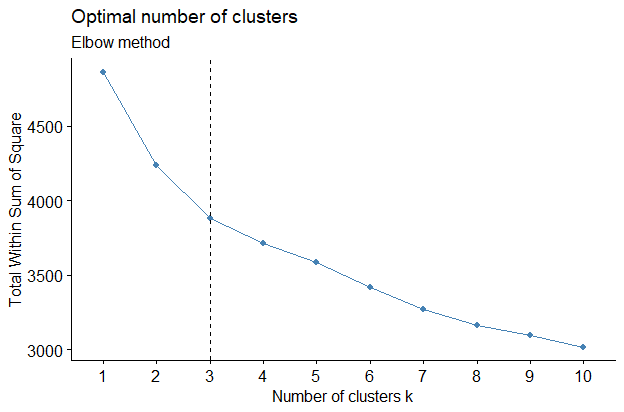 | 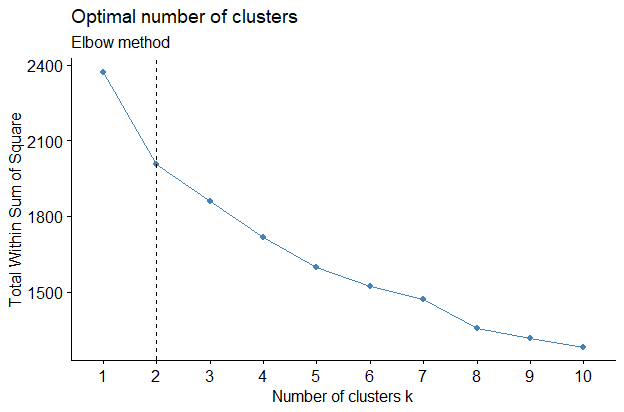 | | 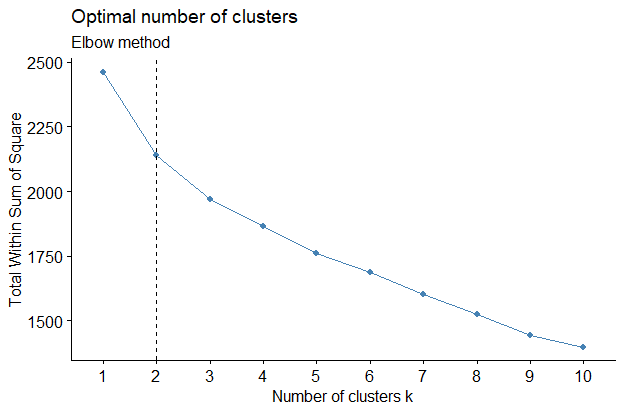 |
| **Gap statistic** | | | |
| 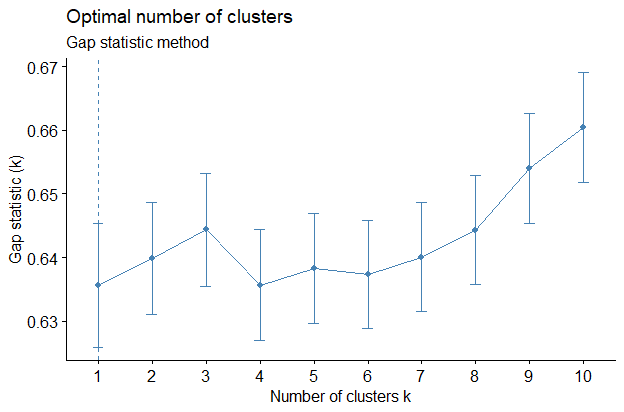 | 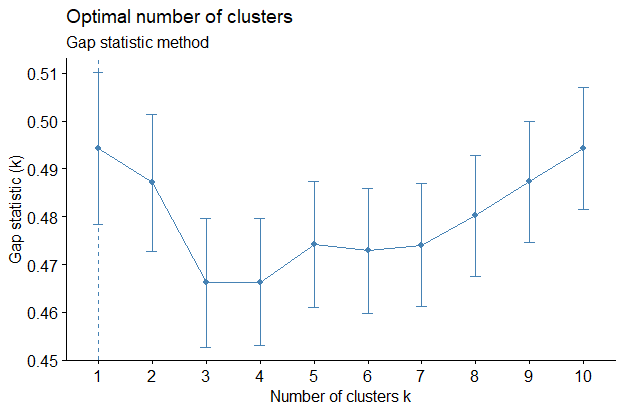 | | 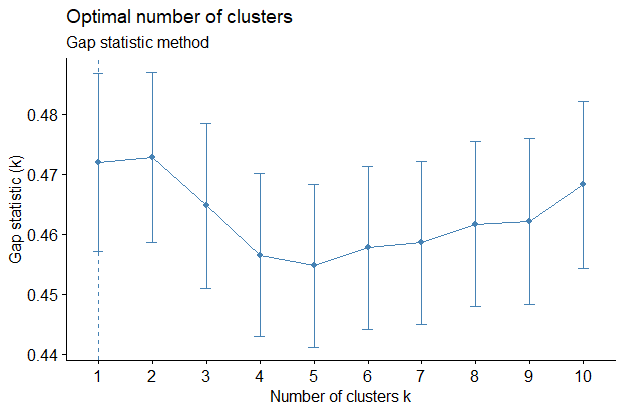 |
| **Silhouette method** | | | |
| 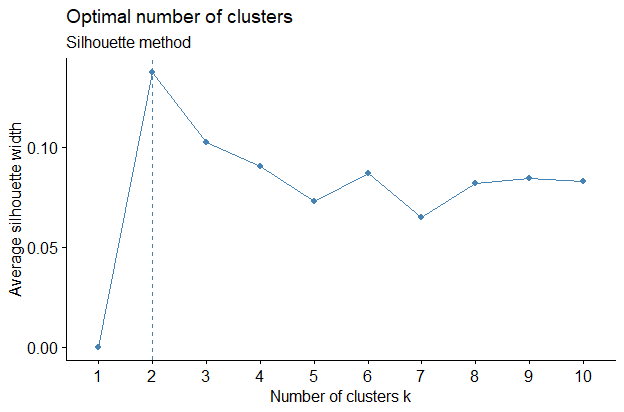 | 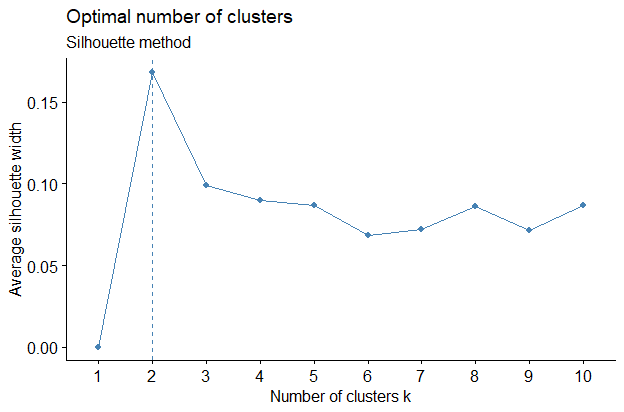 | | 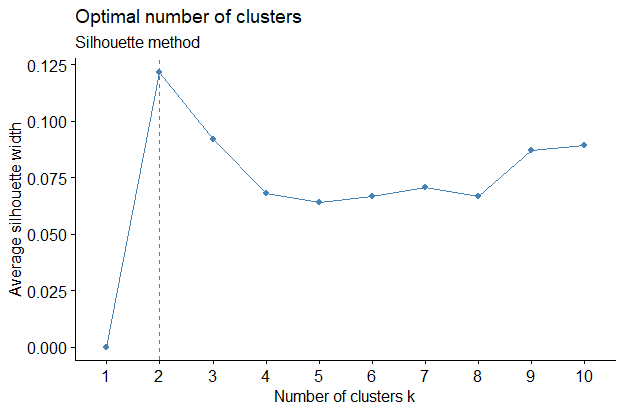 |
| Output from the *NbClust* R package^2^ (all PCPs):  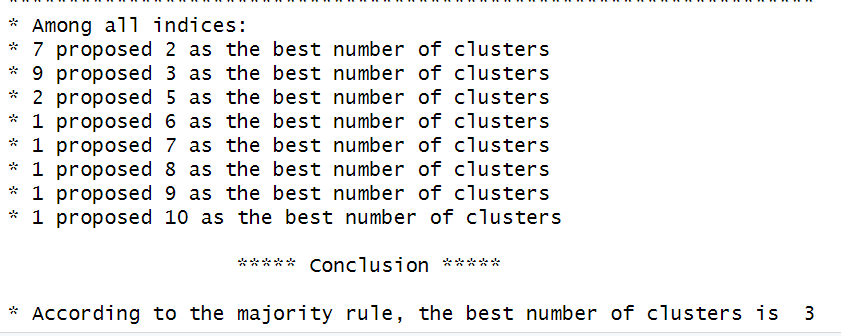 | | | |
| *****Mean panel size: 1,879 patients. | | | |

# eFigure 2. Cluster plots.

| **Part A. All PCPs.** | |
| --- | --- |
| 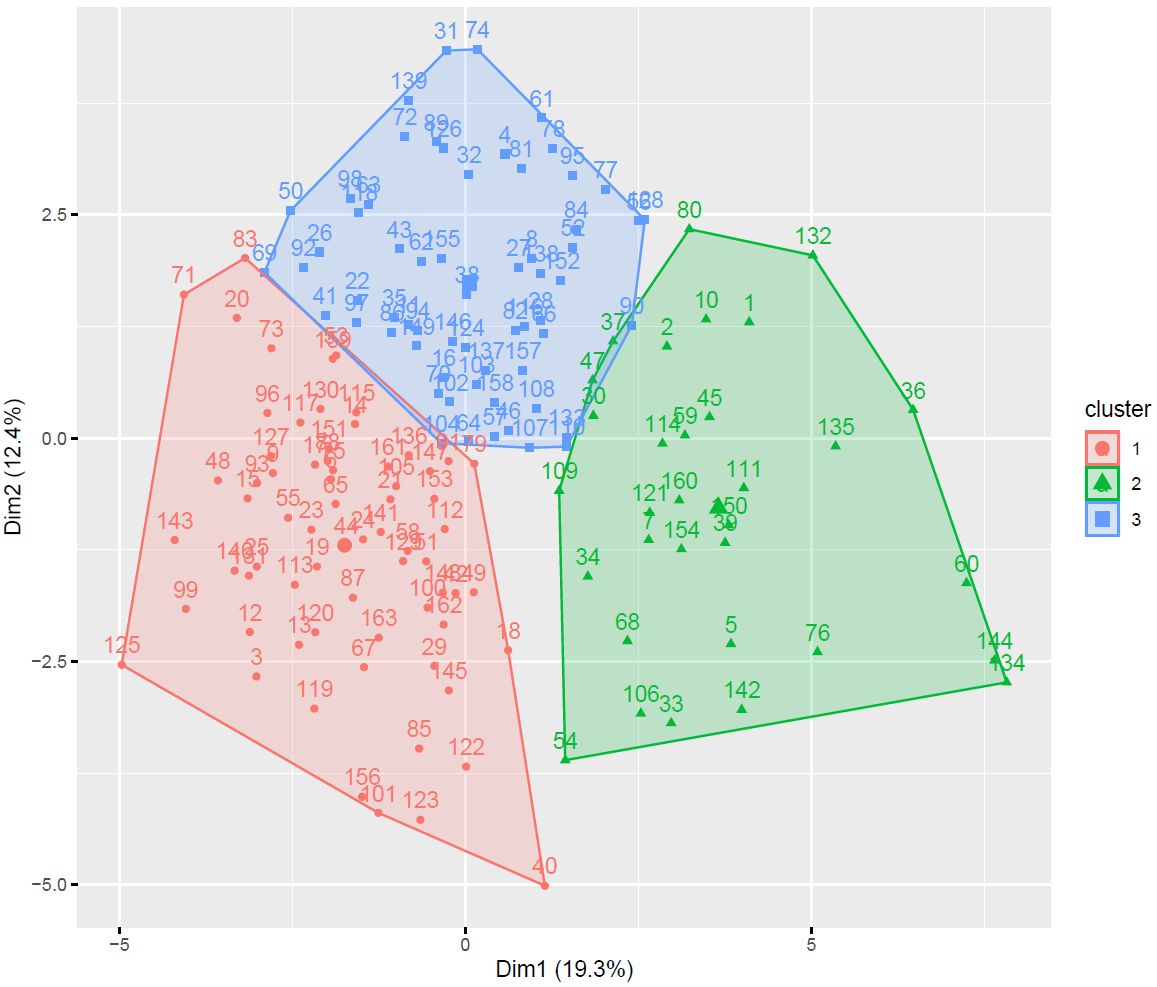 | |
| **Part B. By panel size.** | |
| **<mean panel size*** | **≥mean panel size*** |
| 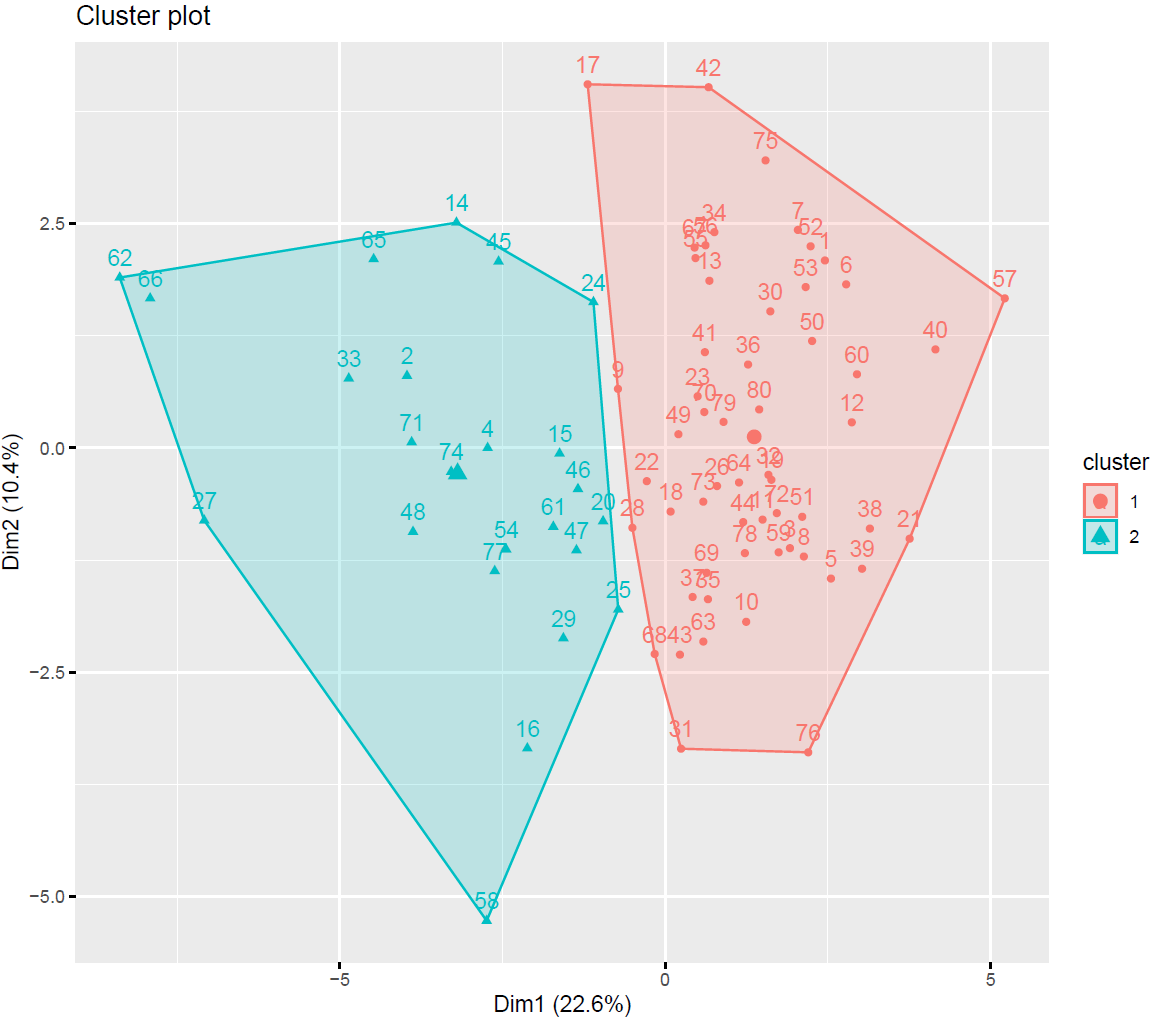 | 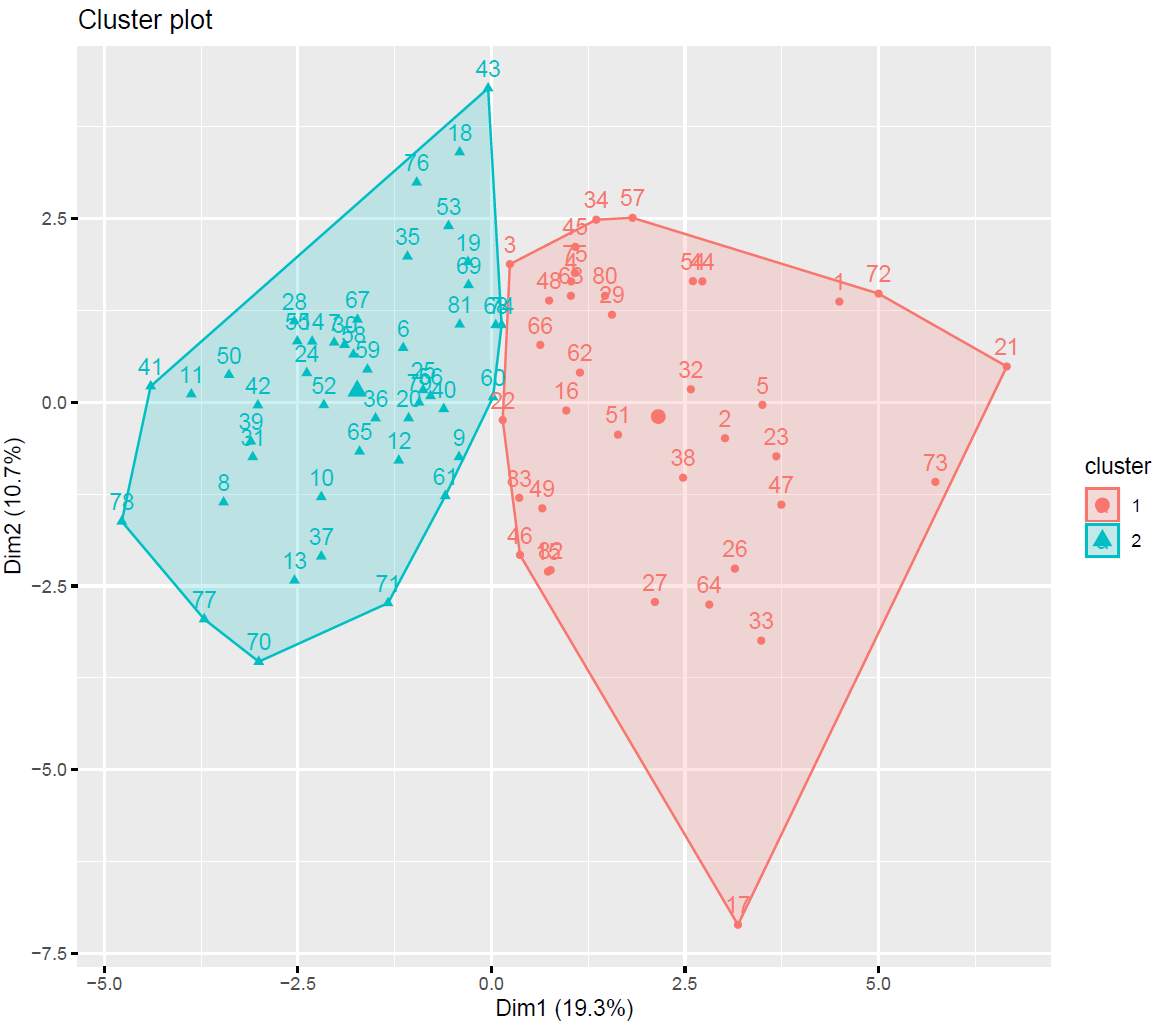 |

The plane in the cluster plot is defined by the first two PCA dimensions │ *Mean panel size: 1,879 patients.

# eTable 2. PCP characteristics and EHR use by cluster.

| **Variable descriptions** |  | **All PCPs** | | | |
| --- | --- | --- | --- | --- | --- |
| **Groups** | |  | | | |
| **Cluster number** | | **1** | **2** | **3** | **SMD** |
|  | **Cluster name** | **High patient load** | **Team-centric workflows** | **Autonomy-driven workflows** |  |
| **Cluster size** | | **62** | **69** | **32** |  |
| **Variables describing PCP and patient characteristics (not used in k-means clustering)** | | | | | |
| Days with scheduled appointments per reporting period, mean % (SD) |  | 0.52 (0.10) | 0.48 (0.12) | 0.50 (0.10) | 0.245 |
| Number of appointments per day with scheduled appointments, mean (SD) |  | 13.59 (2.39) | 10.38 (2.28) | 11.16 (2.00) | 0.945 |
| Clinical full-time equivalent in 2021, mean % (SD) ^1^ |  | 83 (15) | 67 (19) | 66 (21) | 0.613 |
| Admin full-time equivalent in 2021, mean % (SD) ^2^ |  | 3 (8) | 10 (19) | 12 (20) | 0.357 |
| Years of experience working within the healthcare system, mean (SD) |  | 11.83 (9.98) | 14.21 (10.69) | 12.57 (7.63) | 0.164 |
| Provider type, n (%) | Osteopath | 3 (5) | 2 (3) | 1 (3) | 0.067 |
|  | Physician | 59 (95) | 67 (97) | 31 (97) |  |
| Specialty, n (%) | Family Medicine | 7 (11) | 8 (12) | 4 (13) | 0.025 |
|  | Internal Medicine | 55 (89) | 61 (88) | 28 (88) |  |
| Gender, n (%) | Female | 34 (59) | 46 (67) | 23 (72) | 0.236 |
|  | Male | 28 (45) | 23 (33) | 9 (28) |  |
| Race/Ethnicity, n (%) | Asian | 13 (21) | 19 (28) | 9 (28) | 0.19 |
|  | Black | - | 1 (2) | - |  |
|  | Hispanic | - | 6 (9) | 1 (3) |  |
|  | Multiple | - | 1 (2) | - |  |
|  | White | 49 (79) | 42 (61) | 22 (69) |  |
| Panel size in 2021, mean (SD) | | 2239.49 (527.40) | 1589.51 (589.34) | 1808.80 (583.33) | 0.77 |
| Any use of scribes in 2021, n (%) | | 4 (6.5%) | 10 (14.5%) | 4 (12.5%) | 0.176 |
| Patient age (years), mean (SD) | | 57.50 (5.35) | 53.64 (7.80) | 56.81 (5.24) | 0.395 |
| Number of problems on patients' problem lists, mean (SD) | | 12.08 (2.70) | 11.18 (3.64) | 12.75 (2.68) | 0.34 |
| **PCPs’ EHR use data (included in k-means clustering)** | | | | | |
| **General work practices** | | | | | |
| Percentage of days with appointments among days logged into the EHR, mean (SD) | | 67.0 (14.5) | 65.7 (17.0) | 59.0 (12.0) | 0.381 |
| Percentage of patient encounters fully documented and finalized within the same calendar day, mean (SD) | | 0.78 (0.26) | 0.81 (0.22) | 0.63 (0.31) | 0.45 |
| Number of orders placed per day with scheduled appointments, mean (SD) | | 126.24 (23.72) | 80.77 (18.22) | 110.24 (33.10) | 1.27 |
| Number of notes written per day with scheduled appointments, mean (SD) | | 111.21 (24.88) | 61.10 (16.04) | 82.16 (25.12) | 1.519 |
| Number of messages received per day with scheduled appointments, mean (SD) | | 96.83 (21.82) | 62.64 (15.14) | 85.96 (23.72) | 1.156 |
| Percentage of telemedicine visits, mean (SD) | | 0.16 (0.09) | 0.26 (0.13) | 0.19 (0.06) | 0.656 |
| **Collaborative work practices** | | | | | |
| Percentage of orders placed with contributions from other healthcare providers than the PCP, mean (SD) | | 0.07 (0.04) | 0.10 (0.15) | 0.07 (0.04) | 0.222 |
| Percentage of notes written with other sources (e.g., other healthcare providers), mean (SD) | | 0.04 (0.12) | 0.12 (0.25) | 0.02 (0.05) | 0.385 |
| **Time spent in the EHR** |  | | | | |
| Number of minutes in EHR system outside scheduled hours per day with scheduled appointments, mean (SD) | | 58.50 (33.19) | 47.09 (25.72) | 127.64 (37.99) | 1.602 |
| Number of minutes in notes per appointment, mean (SD) | | 7.97 (3.41) | 7.06 (3.35) | 14.14 (4.62) | 1.181 |
| Number of minutes in notes per day on which PCPs accessed the EHR system, mean (SD) | | 69.96 (29.23) | 45.75 (22.81) | 88.22 (25.02) | 1.123 |
| Number of minutes in inbox per appointment,^3^ mean (SD) | | 4.48 (1.47) | 5.46 (2.52) | 8.14 (3.42) | 0.922 |
| Number of minutes in inbox per day on which PCPs accessed the EHR system,^3^ mean (SD) | | 38.51 (11.45) | 33.18 (10.08) | 50.47 (18.32) | 0.816 |
| Number of minutes in orders per appointment, mean (SD) | | 3.90 (0.91) | 3.45 (1.10) | 5.32 (1.24) | 1.122 |
| Number of minutes in orders per day on which PCPs accessed the EHR system, mean (SD) | | 34.30 (8.24) | 22.61 (7.89) | 34.41 (10.03) | 0.923 |
| Number of minutes in clinical review per day on which PCPs accessed the EHR system,^4^ mean (SD) | | 35.27 (11.91) | 30.67 (11.62) | 54.28 (25.62) | 0.843 |
| Number of minutes in clinical review per appointment, mean (SD) | | 3.97 (1.13) | 4.81 (1.86) | 8.48 (3.35) | 1.233 |
| Number of minutes in visit management tool per day on which PCPs accessed the EHR system,^5^ mean (SD) | | 13.70 (4.80) | 9.37 (4.13) | 14.48 (5.72) | 0.714 |
| Number of minutes in EHR on unscheduled days (per day)**,** mean (SD) | | 71.53 (33.95) | 52.58 (23.97) | 149.95 (49.02) | 1.676 |
| Number of minutes accessing the EHR system per day outside of 7am to 7pm, mean (SD) | | 36.89 (19.83) | 23.52 (12.32) | 68.38 (25.86) | 1.464 |
| Turnaround time in days – prescription authorization, mean (SD) | | 0.35 (0.31) | 1.04 (3.06) | 0.70 (0.93) | 0.32 |
| Turnaround time in days – request for obtaining medical advice, mean (SD) | | 2.74 (4.74) | 5.06 (16.27) | 4.91 (9.41) | 0.165 |
| **Use of specific EHR features** | | | | | |
| Percentage of notes written per reporting period using different tools, mean (SD) | EHR shortcuts^6^ | 70.63 (12.93) | 62.14 (13.96) | 63.01 (14.34) | 0.417 |
|  | Copy-paste | 3.96 (6.20) | 5.68 (8.48) | 8.28 (12.93) | 0.298 |
|  | Clinical note editor^7^ | 2.42 (5.78) | 0.78 (3.10) | 1.09 (4.07) | 0.235 |
|  | Manually | 14.10 (8.87) | 21.14 (15.31) | 17.11 (11.30) | 0.386 |
| Percentage of order from order templates & predefined order sets^8^ per reporting period, mean (SD) | | 85.37 (3.54) | 86.69 (3.86) | 86.45 (3.65) | 0.239 |
| Number of action shortcuts^9^ used per day logged into the EHR, mean (SD) | | 2.27 (3.13) | 1.69 (2.26) | 1.05 (1.83) | 0.331 |
| Number of predefined phrases^10^ created by provider per reporting period, mean (SD) | | 140.01 (241.47) | 172.44 (190.10) | 295.92 (364.67) | 0.359 |
| Number of quick diagnosis buttons^11^ created by provider per reporting period, mean (SD) | | 0.76 (0.43) | 0.78 (0.41) | 0.84 (0.37) | 0.138 |
| 1 One full-time equivalent is the workload of one full-time physician. 2 Admin full-time equivalent corresponds to PCPs’ non-clinical workload within the health system. 3 Message center; secure platform for receiving and sending messages.  4 Clinical review: process of examining and assessing clinical information within a patient's electronic record.  5 Visit management tool: structured, customizable workflow tool that guides healthcare providers through the various tasks and documentation steps required during a patient visit.  6 EHR Shortcuts: feature that enhances documentation efficiency by enabling quick insertion of predefined text, data, or templates into notes or records. 7 Documentation tool that guides users through structured, template-based note creation. 8 Predefined groups of orders, documentation templates, and other clinical tools that streamline and standardize the management of specific clinical scenarios or conditions 9 Customizable, one-click shortcuts that automate repetitive tasks. 10 Pre-defined blocks of text or data that can be inserted into a note or documentation with a simple trigger phrase or shortcut. 11 User-defined, customizable quick access buttons. | | | | | |

# eTable 3. PCP characteristics and EHR use by cluster: Stratified by panel size.

| **Variable descriptions** |  | **Panel size** | | | | | |
| --- | --- | --- | --- | --- | --- | --- | --- |
| **Groups** | | **< mean panel size (1,879)** | | | **≥ mean panel size** | | |
| **Cluster number** | | **1** | **2** | **SMD** | **1** | **2** | **SMD** |
|  | **Cluster name** | **Team-centric workflows** | **Autonomy-driven workflows** |  | **Team-centric workflows** | **Autonomy-driven workflows** |  |
| **Cluster size** | | **56** | **24** |  | **46** | **37** |  |
| **Variables describing PCP and patient characteristics (not used in k-means clustering)** | | | | | |  |  |
| Days with scheduled appointments per reporting period, mean % (SD) |  | 0.49 (0.12) | 0.50 (0.11) | 0.14 | 0.48 (0.12) | 0.53 (0.09) | 0.463 |
| Number of appointments per day with scheduled appointments, mean (SD) |  | 9.38 (1.55) | 9.95 (1.07) | 0.424 | 13.84 (1.47) | 13.91 (2.08) | 0.036 |
| Clinical full-time equivalent in 2021, mean % (SD) ^1^ |  | 64 (18) | 59 (18) | 0.278 | 79 (17) | 87 (14) | 0.507 |
| Admin full-time equivalent in 2021, mean % (SD) ^2^ |  | 11 (20) | 15 (22) | 0.169 | 4 (11) | 3 (8) | 0.126 |
| Years of experience working within the healthcare system, mean (SD) |  | 13.77 (10.14) | 12.53 (9.03) | 0.129 | 13.88 (11.52) | 10.96 (7.66) | 0.298 |
| Provider type, n (%) | Osteopath | 1 (2) | - | 0.189 | 3 (7) | 2 (5) | 0.047 |
|  | Physician | 55 (98) | 24 (100) |  | 43 (93) | 35 (95) |  |
| Specialty, n (%) | Family Medicine | 7 (13) | 1 (4) | 0.301 | 5 (11) | 6 (16) | 0.155 |
|  | Internal Medicine | 49 (87) | 23 (96) |  | 41 (89) | 31 (84) |  |
| Gender, n (%) | Female | 40 (71) | 17 (71) | 0.013 | 23 (50) | 23 (62) | 0.244 |
|  | Male | 16 (29) | 7 (29) |  | 23 (50) | 14 (38) |  |
| Race/Ethnicity, n (%) | Asian | 15 (27) | 6 (25) | 0.074 | 10 (22) | 10 (27) | 0.022 |
|  | Black | 1 (2) | - |  | - | - |  |
|  | Hispanic | 2 (4) | 1 (4) |  | 4 (9) | - |  |
|  | Multiple | 1 (2) | - |  | - | - |  |
|  | White | 37 (66) | 17 (71) |  | 32 (69) | 27 (73) |  |
| Panel size in 2021, mean (SD) | | 1511.35 (563.04) | 1680.42 (628.06) | 0.283 | 2171.73 (564.50) | 2203.82 (469.08) | 0.062 |
| Patient age (years), mean (SD) | | 54.33 (7.91) | 57.74 (6.01) | 0.486 | 55.79 (6.28) | 56.48 (5.24) | 0.12 |
| Number of problems on patients' problem lists, mean (SD) | | 11.60 (3.57) | 12.92 (2.76) | 0.415 | 10.89 (2.74) | 12.65 (2.97) | 0.616 |
| **PCPs’ EHR use data (included in k-means clustering)** | |  | | | | | |
| **General work practices** | |  | | | | | |
| Percentage of days with scheduled appointments among EHR access days, mean (SD) | | 67.2 (16.1) | 61.5 (14.3) | 0.371 | 66.0 (18.1) | 62.1 (10.0) | 0.268 |
| Percentage of patient encounters fully documented and finalized within the same calendar day, mean (SD) | | 0.82 (0.20) | 0.64 (0.29) | 0.729 | 0.83 (0.24) | 0.66 (0.30) | 0.617 |
| Number of orders placed per day with scheduled appointments, mean (SD) | | 79.21 (21.15) | 98.25 (23.25) | 0.857 | 114.79 (24.86) | 131.17 (27.53) | 0.624 |
| Number of notes written per day with scheduled appointments, mean (SD) | | 59.54 (16.72) | 77.71 (22.79) | 0.909 | 99.72 (27.48) | 106.85 (29.14) | 0.252 |
| Number of messages received per day with scheduled appointments, mean (SD) | | 62.67 (16.47) | 81.19 (18.77) | 1.049 | 89.34 (25.89) | 94.81 (23.20) | 0.222 |
| Percentage of telemedicine visits, mean (SD) | | 0.26 (0.14) | 0.18 (0.08) | 0.658 | 0.21 (0.11) | 0.17 (0.07) | 0.429 |
| **Collaborative work practices** | |  | | | | | |
| Percentage of orders placed with contributions from other healthcare providers than the PCP, mean (SD) | | 0.09 (0.12) | 0.07 (0.04) | 0.26 | 0.10 (0.13) | 0.07 (0.04) | 0.325 |
| Percentage of notes written with other sources (e.g., other healthcare providers), mean (SD) | | 0.11 (0.24) | 0.04 (0.10) | 0.404 | 0.05 (0.18) | 0.04 (0.13) | 0.062 |
| **Time spent in the EHR** |  |  | | | | | |
| Number of minutes in EHR system outside scheduled hours per day with scheduled appointments, mean (SD) | | 53.84 (25.49) | 127.90 (40.88) | 2.174 | 34.66 (21.71) | 88.68 (36.07) | 1.815 |
| Number of minutes in notes per appointment, mean (SD) | | 7.92 (3.24) | 14.78 (4.66) | 1.709 | 5.45 (2.37) | 10.39 (3.54) | 1.64 |
| Number of minutes in notes per day on which PCPs accessed the EHR system, mean (SD) | | 48.76 (22.30) | 87.58 (28.92) | 1.503 | 49.24 (22.44) | 87.01 (26.68) | 1.532 |
| Number of minutes in inbox per appointment,^3^ mean (SD) | | 5.90 (2.56) | 8.27 (3.38) | 0.792 | 3.99 (1.41) | 5.46 (2.39) | 0.751 |
| Number of minutes in inbox per day on which PCPs accessed the EHR system,^3^ mean (SD) | | 33.92 (10.09) | 48.33 (17.65) | 1.002 | 34.35 (11.39) | 44.66 (14.48) | 0.791 |
| Number of minutes in orders per appointment, mean (SD) | | 3.77 (1.10) | 5.35 (1.33) | 1.292 | 3.17 (0.83) | 4.46 (0.95) | 1.442 |
| Number of minutes in orders per day on which PCPs accessed the EHR system, mean (SD) | | 23.35 (8.45) | 32.19 (10.02) | 0.955 | 28.39 (8.57) | 37.88 (8.30) | 1.124 |
| Number of minutes in clinical review per day on which PCPs accessed the EHR system,^4^ mean (SD) | | 30.33 (11.76) | 45.73 (18.75) | 0.985 | 33.83 (12.24) | 45.63 (23.98) | 0.62 |
| Number of minutes in clinical review per appointment, mean (SD) | | 5.04 (1.90) | 7.85 (3.49) | 0.999 | 3.82 (1.25) | 5.49 (2.89) | 0.753 |
| Number of minutes in visit management tool per day on which PCPs accessed the EHR system,^5^ mean (SD) | | 9.50 (4.09) | 14.86 (6.21) | 1.019 | 11.25 (4.19) | 14.95 (5.10) | 0.794 |
| Number of minutes in EHR on unscheduled days (per day)**,** mean (SD) | | 53.40 (21.23) | 121.73 (55.81) | 1.619 | 52.05 (22.81) | 123.12 (50.23) | 1.822 |
| Number of minutes accessing the EHR system per day outside of 7am to 7pm, mean (SD) | | 25.03 (12.80) | 59.52 (27.46) | 1.61 | 24.85 (14.37) | 57.42 (24.19) | 1.637 |
| Turnaround time in days – prescription authorization, mean (SD) | | 1.20 (3.38) | 0.60 (0.93) | 0.243 | 0.35 (0.23) | 0.49 (0.52) | 0.355 |
| Turnaround time in days – request for obtaining medical advice, mean (SD) | | 6.19 (18.13) | 4.81 (10.39) | 0.094 | 1.69 (1.70) | 3.68 (5.53) | 0.487 |
| **Use of specific EHR features** | |  | | | | | |
| Percentage of notes written per reporting period using different tools, mean (SD) | EHR shortcuts^6^ | 62.69 (13.89) | 61.46 (15.20) | 0.085 | 69.67 (13.68) | 67.36 (13.26) | 0.172 |
|  | Copy-paste | 5.45 (8.48) | 9.78 (13.61) | 0.382 | 2.90 (4.00) | 6.18 (9.30) | 0.458 |
|  | Clinical note editor ^7^ | 0.83 (2.89) | 0.80 (2.38) | 0.012 | 1.39 (4.17) | 2.95 (7.10) | 0.269 |
|  | Manually | 21.54 (14.93) | 19.19 (11.02) | 0.179 | 14.67 (11.99) | 14.56 (9.31) | 0.01 |
| Percentage of order from order templates & predefined order sets^8^ per reporting period, mean (SD) | | 86.23 (4.15) | 86.38 (2.87) | 0.041 | 86.03 (3.92) | 85.99 (3.40) | 0.011 |
| Number of action shortcuts^9^ used per day logged into the EHR, mean (SD) | | 1.71 (2.34) | 1.56 (2.51) | 0.06 | 1.87 (2.91) | 1.94 (2.64) | 0.023 |
| Number of predefined phrases^10^ created by provider per reporting period, mean (SD) | | 185.72 (196.95) | 431.91 (495.65) | 0.653 | 80.88 (90.05) | 150.31 (119.22) | 0.657 |
| Number of quick diagnosis buttons^11^ created by provider per reporting period, mean (SD) | | 0.75 (0.44) | 0.91 (0.28) | 0.444 | 0.80 (0.40) | 0.73 (0.45) | 0.158 |
| 1 One full-time equivalent is the workload of one full-time physician. 2 Admin full-time equivalent corresponds to PCPs’ non-clinical workload within the health system. 3 Message center; secure platform for receiving and sending messages.  4 Clinical review: process of examining and assessing clinical information within a patient's electronic record.  5 Visit management tool: structured, customizable workflow tool that guides healthcare providers through the various tasks and documentation steps required during a patient visit.  6 EHR shortcuts: feature that enhances documentation efficiency by enabling quick insertion of predefined text, data, or templates into notes or records. 7 Documentation tool that guides users through structured, template-based note creation. 8 Predefined groups of orders, documentation templates, and other clinical tools that streamline and standardize the management of specific clinical scenarios or conditions 9 Customizable, one-click shortcuts that automate repetitive tasks. 10 Pre-defined blocks of text or data that can be inserted into a note or documentation with a simple trigger phrase or shortcut. 11 User-defined, customizable quick access buttons. | | | | | | | |

| eFigure 3. Relative differences in PCPs’ EHR use by cluster (*compared to Cluster 2*) |
| --- |
| *Cluster sizes:* Cluster 1 “High patient load”: n=62 │Cluster 2 “Team-centric workflows”: n=69 (base cluster) │Cluster 3 “Autonomy-driven workflows”: n=32 |
| 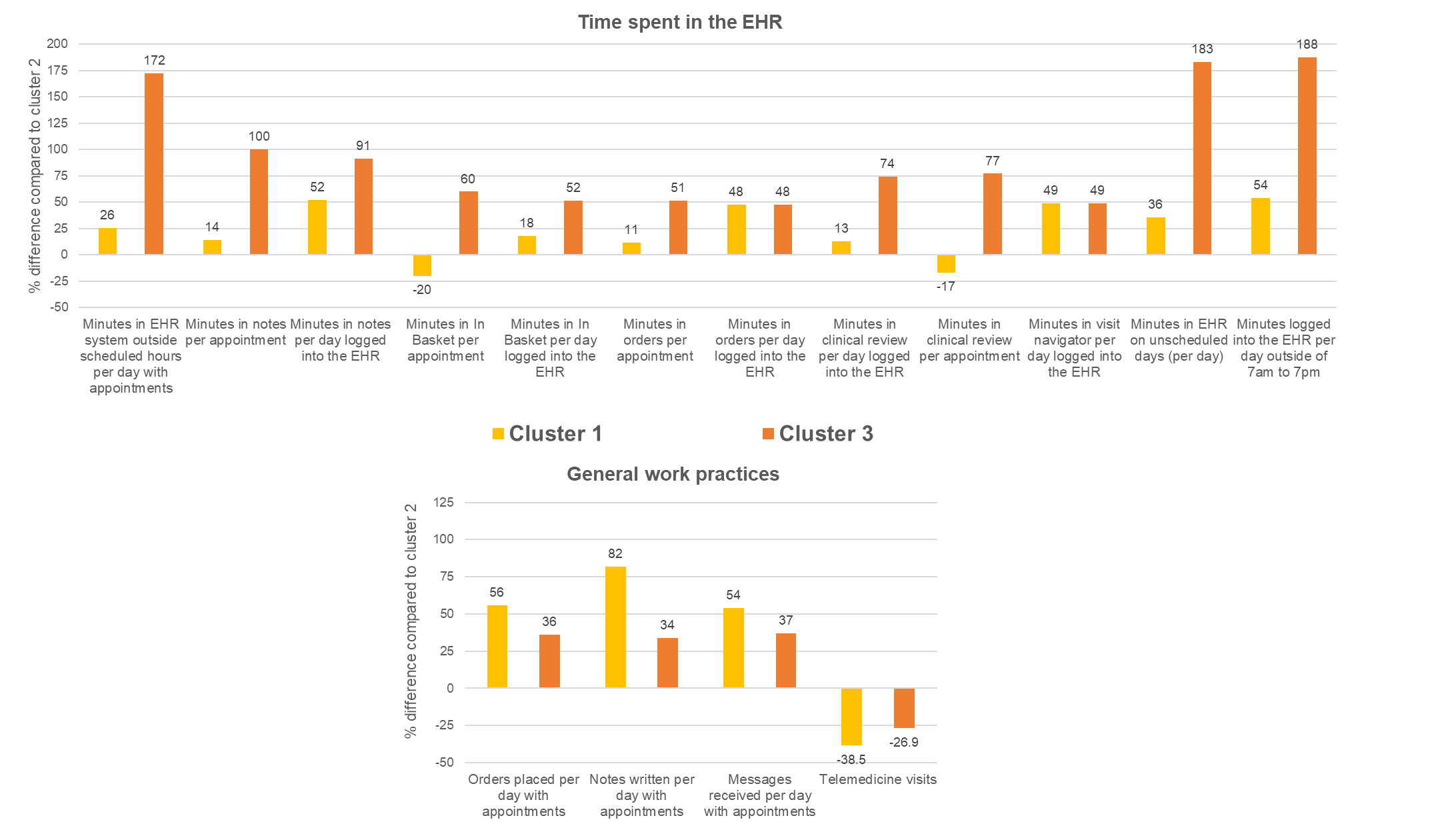 |
| Only variables with standardized mean differences (SMD) ≥0.5 and variables included in the k-means clustering presented here. |

# eFigure 4. Differences in PCPs’ EHR use by cluster: Stratified by panel size (relative differences)

| *Relative differences compared to cluster 1 of each stratified analysis* |
| --- |
| 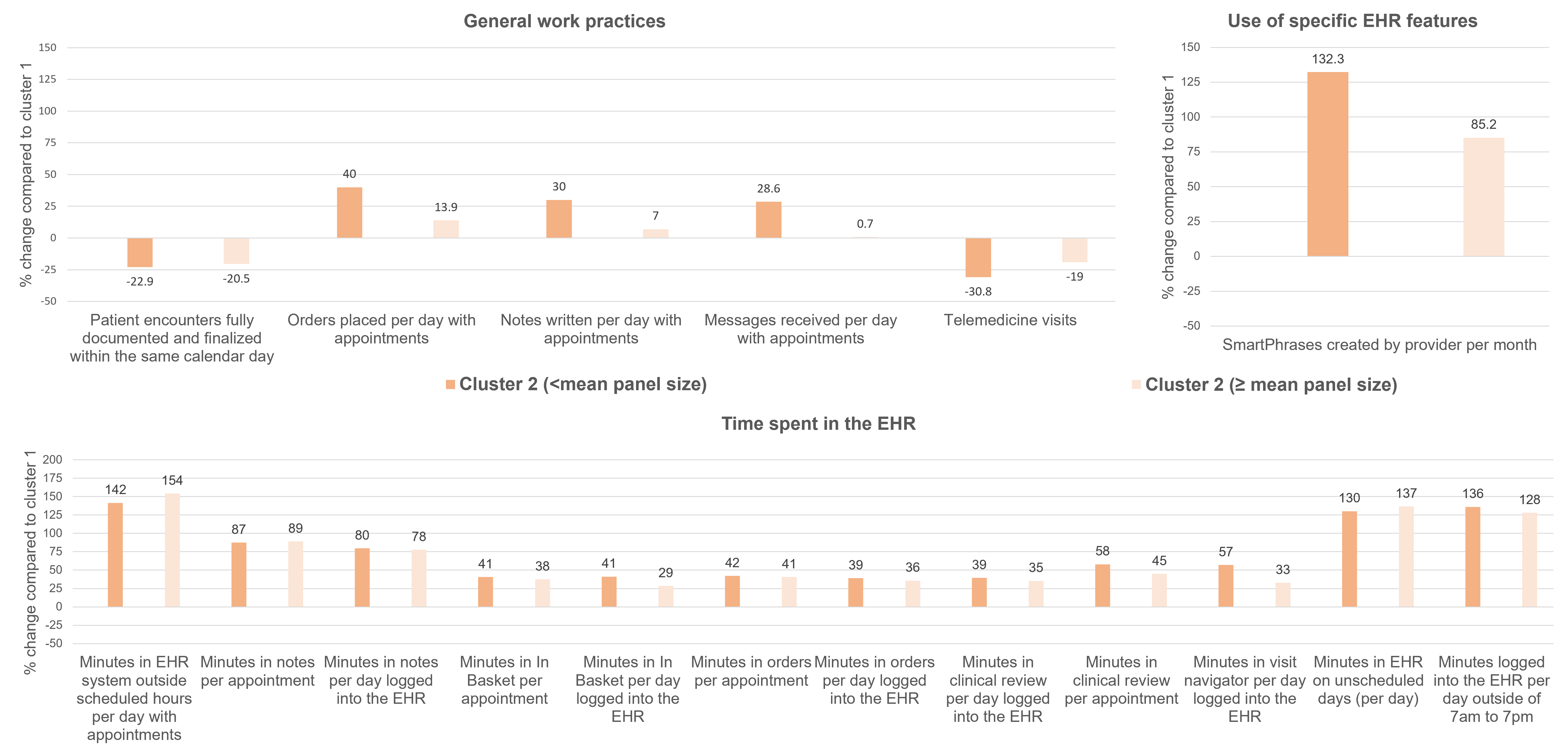 |
| *Only variables with standardized mean differences (SMD) ≥0.5 and those included in the k-means clustering presented here │ Mean panel size: 1,879 patients |

## eTable 4. Multinomial regression results of the associations between PCP characteristics and the identified EHR use clusters (n=163)

| **Variable Name** | **Relative Risk** | **95% Confidence Interval** |
| --- | --- | --- |
| **Base cluster: Cluster 2 (Team-centric workflows)** |  |  |
| **Cluster 1 (High patient load)** |  |  |
| Average numbers of problems on patients’ problem lists (per 1 appointment) | 1.07 | 0.96 to 1.19 |
| Average number of days with appointments (per 1-percent increase) | 2.35 | 0.23 to 24.07 |
| Administrative duties based on FTE (reference: no admin duties based on FTE) | 0.33 | 0.13 to 0.82* |
| Average number of years within healthcare system (Atrius) (per 10-year increase) | 0.55 | 0.36 to 0.83* |
| Provider type: Osteopath (reference: physician) | 1.01 | 0.15 to 6.67 |
| Specialty: Family Medicine (reference: Internal Medicine) | 0.56 | 0.17 to 1.85 |
| Male gender (reference: female) | 1.50 | 0.69 to 3.27 |
| Non-White race/ethnicity^1^ (reference: White) | 0.31 | 0.14 to 0.72* |
| **Cluster 3 (Autonomy-driven workflows)** |  |  |
| Average numbers of problems on patients’ problem lists (per 1 appointment) | 1.00 | 0.98 to 1.24 |
| Average number of days with appointments (per 1-percent increase) | 0.21 | 0.01 to 3.10 |
| Admin FTE (reference: no admin FTE) | 0.77 | 0.30 to 2.01 |
| Average number of years within healthcare system (Atrius) (per 10-year increase) | 0.63 | 0.39 to 1.03 |
| Provider type: Osteopath (reference: physician) | 0.74 | 0.06 to 8.89 |
| Specialty: Family Medicine (reference: Internal Medicine) | 0.76 | 0.20 to 2.93 |
| Male gender (reference: female) | 0.69 | 0.27 to 1.81 |
| Non-White race/ethnicity* (reference: White) | 0.52 | 0.21 to 1.31 |

^1^ Asian, Black, Hispanic, or Multiple races/ethnicities │* p-value < 0.05

**References**

1. UC Business Analytics R Programming Guide. K-means Cluster Analysis. <https://uc-r.github.io/kmeans_clustering>

2. Charrad M GN, Boiteau V, Niknafs A (2014). “NbClust: An R Package for Determining the Relevant Number of Clusters in a Data Set.” Journal of Statistical Software, 61(6), 1–36. <https://www.jstatsoft.org/v61/i06/>.

3. RDocumentation. kmeans: K-Means Clustering. <https://www.rdocumentation.org/packages/stats/versions/3.6.2/topics/kmeans>

4. Towards Machine Learning. What is K-Means algorithm and how it works. <https://towardsmachinelearning.org/k-means/>
